# Detecting zoonotic Influenza A using QIAstat-Dx Respiratory SARS-CoV-2 panel for pandemic preparedness

**DOI:** 10.1038/s41598-023-29838-9

**Published:** 2023-02-17

**Authors:** Luis Peñarrubia, Sonia N. Rao, Roberto Porco, Marta Varo, Patricia Muñoz-Torrero, Fernando Ortiz-Martinez, Josep Pareja, Marta López-Fontanals, Davide Manissero

**Affiliations:** 1 STAT-Dx Life S.L. (A QIAGEN Company), Baldiri Reixac, 4, 08028 Barcelona, Spain; 2 QIAGEN Inc., 19300 Germantown Road, Germantown, MD 20874 USA; 3 QIAGEN Manchester Ltd, Skelton House, Lloyd Street North, Manchester, M15 6SH UK

**Keywords:** Molecular biology, Infectious diseases, Influenza virus

## Abstract

Recent reports from the World Health Organization regarding Influenza A cases of zoonotic origin in humans (H1v and H9N2) and publications describing emergence swine Influenza A cases in humans together with “G4” Eurasian avian-like H1N1 Influenza A virus have drawn global attention to Influenza A pandemic threat. Additionally, the current COVID-19 epidemic has stressed the importance of surveillance and preparedness to prevent potential outbreaks. One feature of the QIAstat-Dx Respiratory SARS-CoV-2 panel is the double target approach for Influenza A detection of seasonal strains affecting humans using a generic Influenza A assay plus the three specific human subtype assays. This work explores the potential use of this double target approach in the QIAstat-Dx Respiratory SARS-Co-V-2 Panel as a tool to detect zoonotic Influenza A strains. A set of recently recorded H9 and H1 spillover strains and the G4 EA Influenza A strains as example of recent zoonotic Flu A strains were subjected to detection prediction with QIAstat-Dx Respiratory SARS-CoV-2 Panel using commercial synthetic dsDNA sequences. In addition, a large set of available commercial human and non-human influenza A strains were also tested using QIAstat-Dx Respiratory SARS-CoV-2 Panel for a better understanding of detection and discrimination of Influenza A strains. Results show that QIAstat-Dx Respiratory SARS-CoV-2 Panel generic Influenza A assay detects all the recently recorded H9, H5 and H1 zoonotic spillover strains and all the G4 EA Influenza A strains. Additionally, these strains yielded negative results for the three-human seasonal IAV (H1, H3 and H1N1 pandemic) assays. Additional non-human strains corroborated those results of Flu A detection with no subtype discrimination, whereas human Influenza strains were positively discriminated. These results indicate that QIAstat-Dx Respiratory SARS-CoV-2 Panel could be a useful tool to diagnose zoonotic Influenza A strains and differentiate them from the seasonal strains commonly affecting humans.

## Introduction

One important public health lesson learned from the Severe Acute Respiratory Syndrome Coronavirus 2 (SARS-CoV-2) pandemic has been the importance of time to action and preparedness for detection of novel viruses with pandemic potential. Indeed, humans can be infected with zoonotic influenza viruses, such as avian influenza virus subtypes A(H5N1), A(H7N9), and A(H9N2) and swine influenza virus subtypes A(H1N1), A(H1N2) and A(H3N2). The most likely explanation for the appearance of new pandemic strains in humans is the reassortment between currently circulating human strains and avian influenza viruses, or by direct transfer^1–3^. Examples of zoonotic Flu A strains can be extracted from representative literature recently available. The epidemiological record from the World Health Organization (WHO)^4^ highlights that recent human cases of swine-originated Influenza A (H1)v have been recorded in Germany (H1N1), Brazil (H1N2) and the Netherlands (H1N1), as well as five human cases of avian Influenza A (H9N2) detected in China. WHO also reported the first case of avian influenza A (H5N8) detection in seven human clinical specimens in Russia^5^. In addition to these recorded swine and avian Influenza A spillovers, a recent publication in the *Proceedings of the National Academy of Science* by Sun H. and colleagues described the predominant genotype “G4” Eurasian (EA) avian-like H1N1 virus that has been spreading among pigs in China since 2016, which has H1N1 pandemic 2009 (H1N1pdm09) and triple-reassortant (TR)-derived internal genes^6^. Similar observations have been previously reported by Henritzi et al.^7^. Authors demonstrated that intensive reassortment with human pandemic H1N1pdm09 virus produced large number of hemagglutinin/neuraminidase combinations that could derivate in changes in virulence. It is well established that efficient human-to-human transmission is a critical feature of pandemic influenza viruses^8,9^. Sun et al. determined that the emergent novel G4 reassortment EA H1N1 virus has high infectivity and transmissibility potential via direct contact and respiratory droplets amongst ferrets, suggesting ability to infect humans similar to the H1 pandemic in 2009^7,10^. Additionally, the reported serological surveillance amongst swine workers with occupational exposure showed 10.4% were positive for G4 swine EA H1N1 with younger 18–35-year-old patients demonstrating higher rates of seropositivity. These data caught the attention of the US Center for Disease Control and Prevention which has already determined that their current Influenza Virus Retrotranscriptase Quantitative PCR (RT-qPCR) Influenza A (H1/H3/H1N1pdm09) Subtyping panel would detect this swine influenza virus (SIV)^11^. This would yield a positive result for the subtype H1N1, which would indicate that the H1N1 subtype assay shows cross-reactivity to swine variants of the H1 genotype.

The overlap of viral etiologies and clinical presentation of Influenza-Like Illness (ILI) during the seasonal influenza period has increased the attention to syndromic diagnostics with multiplex capability, as well as the need to improve ability to detect and distinguish new variants. The QIAstat-Dx Respiratory SARS-CoV-2 Panel offers a multiplex RT-qPCR platform detecting and discriminating 22 targets (CE-IVD) or 21 targets (FDA EUA) as one of the currently available respiratory syndromic solutions^12^. The result provided by this panel would allow a differential diagnosis of pathogens (18 viruses and 4 atypical bacteria) that share overlapping ILI symptoms.

One feature of the QIAstat-Dx Respiratory SARS-CoV-2 panel is the double target approach to Influenza A detection. Specifically, this detection is based on a generic assay designed to amplify any Influenza A strain, as well as three specific assays to discriminate between the three main Influenza A Virus (IAV) subtypes infecting humans: seasonal IAV H1, seasonal IAV H3, and IAV H1N1pdm09 among screening of the M, HA and NA targeted genes. The mentioned generic Influenza A design targets the Matrix gene which is one with highest homology among IAV. The dual target approach enhances the detection of the IAV genotypes affecting humans, while allowing the amplification of novel strains by detecting Influenza A targets without human subtype (ruling out H1, H3 and H1N1pdm09).

Influenza A seasonal H1 subtype (derived from the 1977 lineage)^2,3^, was co-circulating with other lineages until 2009^8^. By this year, a third lineage (H1N1pdm09) derived from the triple-reassortant swine H1N2 lineage^3^ has displaced seasonal H1. Although seasonal H1 subtype is not in circulation, the QIAstat-Dx Respiratory SARS-CoV-2 Panel contains primers and probes to detect all three human subtypes. This strategy allows to discriminate strains with a genetic variability close to H1N1pdm09 cluster with zoonotic origin but still not H1N1pdm09.

The QIAstat-Dx Respiratory SARS-CoV-2 Panel detects all HA lineages (1A, 1B and 1C corresponding to classical swine lineage; human seasonal lineage and Eurasian avian lineage) including all subclusters^9^ and only differentiates among human H1 (seasonal and pandemic separately) subtypes. This information is essential for further epidemiologic studies and phylogenetic analyses to properly characterize and monitor Influenza A spread. After detecting presence of any Influenza A strain in the tested sample, further analyses are needed to correctly classify the Flu A strain based on the global Influenza A nomenclature^9^.

In this work we sought to understand the detectability of the recently recorded zoonotic spillovers Influenza A strains and the potentially pandemic G4 EA swine Influenza A strain using QIAstat-Dx Respiratory SARS-CoV2 Panel. To do so, an initial *in-silico* analysis of all available sequences was performed for the selection of final strains to be tested for specificity in the laboratory. Based on the lack of commercial available strains for the serotypes analyzed in this study, an *in-vitro* confirmation was performed using commercial synthetic dsDNA sequences emulating the viral nucleic acids of the 4 target regions of the selected Influenza A strains genome detected by QIAstat-Dx Respiratory SARS-CoV-2 Panel.

## Materials and methods

A thorough bioinformatic screening was performed to characterize the possible amplification of some Influenza A (H1)v and H9N2 described as candidate vaccine viruses (CVVs) in the Epidemiological Record by the WHO^4^, in addition to H5N8 genome sequences collected from the first reported detection in humans during December, 2020 in Russia (www.who.int/csr/don/26-feb-2021-influenza-a-russian-federation/en/), and also the G4 swine EA H1N1 pdm09-like lineage^6^.

Based on WHO report^4^, five human cases of A(H9N2) virus infection were detected from 25 February to 30 September 2020 corresponding to Y280/G9-lineage, and four of them were sequenced, showing HA genes with greatest similarity to the A/Anhui-Lujiang/39/2018 and the A/Hong Kong/308/2014 isolates. In addition, two cases of A(H1N1)v human infection identified in Germany in the Netherlands were identified in the same period of time, and sequences for those A/Hessen/47/2020 and A/Netherlands/3315/2016 lineages^4^ were included in the analysis. Genome sequences from those four lineages were collected from GISAID database^13^.

In addition, genomic fragments corresponding to the Influenza A/Astrakhan/3212/2020 (H5N8) corresponding to the first reported detection in humans in Russia ^5^ were also included. Genome was collected from GISAID database with Accesion ID EPI_ISL_1038924. Finally, the entire set of sequence accessions of the 29 G4 strains described in Sun et al.^6^ were downloaded from GenBank (NCBI) data base, including the eight fragments of every screened genome. All accessions used in this study are listed in Supplementary file 1.

An alignment between the different segments of all collected viral genomes and the Influenza A RT-qPCR assays targeted by the QIAstat-Dx Respiratory SARS-CoV-2 panel was carried out using ClustalW algorithm implemented in Geneious software v.10.2.6 (http://www.geneious.com), with a gap open and extent cost of 5 and 3 respectively. The output alignment was curated manually.

In order to obtain a final list of strains to be tested in laboratory, a preliminary specificity assessment on the criticality of the mismatches between the viral sequences and the oligonucleotide sequences included in the QIAstat-Dx Respiratory Panel was made to establish a prediction on the potential amplification of the targeted regions of the selected (H1)v, H5N8 and H9N2 viral genomes. Every screened primer set (general Influenza A detection; together with H1, H3 and H1N1pdm09 subtypes) was considered unspecific for the analyzed sequences if: 1) three or more mismatches were found among any individual oligonucleotide sequence and the strain gene sequences or 2) variations were placed in the three last nucleotides of the 3’-end or in the 5’-end of the probe affecting the PCR amplification^14–16^. This approach has been used previously to characterize inclusivity of specific RT-qPCR assays for viral detection^17^. Sequences predicted to be negative were considered as negatively detected and they were discarded from the analysis.

Laboratory testing was performed to confirm *in-silico* prediction for positive detected strains. A total of 31 gBlocks (dsDNA fragments of 500 bp size) were designed to cover all the genetic variability observed in the targeted segments among the analyzed genomes (see Supplementary file 2). The four strains collected from GISAID data base corresponding to zoonotic H1N1 and H9N2 viruses detected in humans were represented among nine gBlocks covering all QIAstat-Dx Respiratory SARS-CoV-2 targeted genomic segments, whereas the remaining 22 gBlocks were designed to cover the 29 G4 EA sequences described in Sun et al.^6^. Based on mutations observed in sequence alignment, all those gBlocks were mixed accordingly in 17 unique combinations (tested in a single replicate at 1E + 05 copies/mL concentration to ensure specificity) in order to simulate all the genetic variability of the viral genomic regions targeted by the QIAstat-Dx Respiratory SARS-CoV-2 Panel. Finally, two analytical samples were also included in the analysis as controls corresponding to a known seasonal H1 and H1N1pdm09 human strains (Table 1).

**Table 1:** Detection of zoonotic Influenza A strains by the QIAstat-Dx Respiratory SARS-CoV-2 Panel.

Source	Flu A strain		gBlocks Combination	QIAstat-Dx Generic Influenza A detection		QIAstat-Dx Seasonal H1 subtype detection		QIAstat-Dx H1N1pdm09 subtype detection	
	Strain number	Strain name		Ct	EPF	Ct	EPF	Ct	EPF
swine “G4” Eurasian (EA) avian-like H1N1 Influenza A virus ^6^	1	A/swine/Anhui/0202/2018	#1	30.2	109,294	Neg	Neg	Neg	Neg
	2	A/swine/Anhui/0203/2018	#1	30.2	109,294	Neg	Neg	Neg	Neg
	3	A/swine/Beijing/0301/2018	#2	30.1	143,172	Neg	Neg	Neg	Neg
	4	A/swine/Hebei/0113/2017	#3	30.3	67,620	Neg	Neg	Neg	Neg
	5	A/swine/Hebei/0116/2017	#4	29.5	67,145	Neg	Neg	Neg	Neg
	6	A/swine/Hebei/0221/2017	#5	28.5	45,229	Neg	Neg	Neg	Neg
	7	A/swine/Heilongjiang/0110/2017	#6	30.3	71,669	Neg	Neg	Neg	Neg
	8	A/swine/Heilongjiang/0140/2017	#7	32.2	88,997	Neg	Neg	Neg	Neg
	9	A/swine/Heilongjiang/1214/2016	#6	30.3	71,669	Neg	Neg	Neg	Neg
	10	A/swine/Henan/SN10/2018	#8	30.9	96,817	Neg	Neg	Neg	Neg
	11	A/swine/Henan/SN11/2018	#8	30.9	96,817	Neg	Neg	Neg	Neg
	12	A/swine/Henan/SN12/2018	#8	30.9	96,817	Neg	Neg	Neg	Neg
	13	A/swine/Henan/SN13/2018	#8	30.9	96,817	Neg	Neg	Neg	Neg
	14	A/swine/Jiangsu/J004/2018	#1	30.2	109,294	Neg	Neg	Neg	Neg
	15	A/swine/Jiangsu/J005/2018	#1	30.2	109,294	Neg	Neg	Neg	Neg
	16	A/swine/Jiangsu/J006/2018	#1	30.2	109,294	Neg	Neg	Neg	Neg
	17	A/swine/Jilin/21/2016	#9	30.5	149,619	Neg	Neg	Neg	Neg
	18	A/swine/Jilin/23/2016	#9	30.5	149,619	Neg	Neg	Neg	Neg
	19	A/swine/Jilin/29/2016	#9	30.5	149,619	Neg	Neg	Neg	Neg
	20	A/swine/Shandong/0334/2017	#10	29.0	92,841	Neg	Neg	Neg	Neg
	21	A/swine/Shandong/0336/2017	#6	30.3	71,669	Neg	Neg	Neg	Neg
	22	A/swine/Shandong/0337/2017	#6	30.3	71,669	Neg	Neg	Neg	Neg
	23	A/swine/Shandong/1203/2016	#11	30.2	75,277	Neg	Neg	Neg	Neg
	24	A/swine/Shandong/1207/2016	#6	30.3	71,669	Neg	Neg	Neg	Neg
	25	A/swine/Shandong/16/2016	#9	30.5	149,619	Neg	Neg	Neg	Neg
	26	A/swine/Shandong/36/2016	#9	30.5	149,619	Neg	Neg	Neg	Neg
	27	A/swine/Shandong/9/2016	#9	30.5	149,619	Neg	Neg	Neg	Neg
	28	A/swine/Shandong/JM78/2017	#12	30.3	117,056	Neg	Neg	Neg	Neg
	29	A/swine/Shandong/LY142/2017	#13	30.8	120,913	Neg	Neg	Neg	Neg
2020 candidate vaccine viruses (CVVs) ^5^	30	A/Hessen/47/2020|H1N1	#14	31.0	137,759	Neg	Neg	Neg	Neg
	31	A/Netherlands/3315/2016|H1N1	#15	28.5	94,076	Neg	Neg	Neg	Neg
	32	A/Anhui-Lujiang/39/2018|H9N2	#16	31.6	124,115	Neg	Neg	Neg	Neg
	33	A/Hong_Kong/308/2014|H9N2	#17	29.9	148,953	Neg	Neg	Neg	Neg
Controls^1^	C1	A/H1N1/Brisbane/59/07	n/a	14.9	190,941	15.0	81,471	Neg	Neg
	C2	A/Virginia/ATCC1/2009	n/a	15.3	188,178	Neg	Neg	18.0	41,804

A set of recently reported H9, H1 spillover, and the G4 EA Influenza A strains were detected using the QIAstat-Dx Respiratory SARS-CoV-2 Panel, using commercial synthetic gBlock dsDNA sequences. All the recently recorded H9 and H1 zoonotic spillover strains and all the G4 EA Influenza A strains were successfully detected. Negative results for three human seasonal IAV’s (H1, H3 and H1N1 pandemic) indicate a high specificity in the detection assay.

*EPF* End-point-fluorescence. *Neg* Negative result.

^1^C1 and C2 correspond to Control 1 (Seasonal human H1 subtype; Zeptometrix, Catalogue ID: 0,810,244) and Control 2 (H1N1pdm09 human subtype; ATCC, Catalogue ID: VR-1736).

Secondly, a large set of additional human and non-human Influenza A strains were also tested by the QIAstat-Dx Respiratory SARS-CoV-2 Panel to enlarge inclusivity list of strains tested and to give more clarity on the importance of Cycle Threshold (Ct) correlation obtained during RT-qPCR in the QIAstat-Dx system between generic Flu A assay and all three Flu A subtypes assays for the discrimination of non-human Flu A strains. A total of 19 common human Influenza A strains (seven seasonal H1, six seasonal H3 and 6 H1N1pdm09) and genomic material for four non-human strains available from commercial suppliers (ATCC, Manassas, Virginia; Zeptometrix, Buffalo, NY) were tested in triplicates for two consecutive ten-fold dilutions (see Table 2) in low concentrations to characterize their detection and correct subtype discrimination. Dilutions have been selected according to sensitivity level of the QIAstat-Dx Respiratory SARS-CoV-2 panel described in the Instructions For Use^12^. Additional information of the panel performance as the use of the Internal Control and interpretation of results is also described.

**Table 2:** Detection of some human and non-human Influenza A strains by the QIAstat-Dx Respiratory SARS-CoV-2 Panel.

Type	Name (serotype)	ID (supplier)	Concentration tested	QIAstat-Dx generic Influenza A Ct (SD)	QIAstat-Dx Influenza A H1 Subtype Ct (SD)	QIAstat-Dx Influenza A H3 Subtype Ct (SD)	QIAstat-Dx Influenza A H1N1pdm09 subtype Ct (SD)
H1N1 subtype	A/Denver/1/57	VR-546 (ATCC	3.4E + 03 CEID50/mL	33.5 (0.2)	33.1 (0.8)	Neg	Neg
			3.4E + 02 CEID50/mL	36.1 (0.1)	36.8 (1.0)	Neg	Neg
	A/Mal/302/54	VR-98 (ATCC)	1.6E + 02 CEID50/mL	32.3 (0.8)	30.9 (0.2)	Neg	Neg
			1.6E + 01 CEID50/mL	35.2 (0.2)	34.4 (0.5)	Neg	Neg
	A/Weiss/43	VR-96 (ATCC)	2.8E + 04 CEID50/mL	37.1 (0.8)	38.0 (0.3)	Neg	Neg
			2.8E + 03 CEID50/mL	37.9 (0.2)	38.0 (0.3)	Neg	Neg
	A/PR/8/34	VR-1469 (ATCC)	3.9E + 02 PFU/mL	34.4 (0.8)	33.7 (0.1)	Neg	Neg
			3.9E + 01 PFU/mL	37.7 (0.12)	37.5 (0.9)	Neg	Neg
	A/Fort Monmouth/1/1947	VR-1754 (ATCC)	2.8E + 03 CEID50/mL	32.6 (1.0)	31.5 (0.6)	Neg	Neg
			2.8E + 02 CEID50/mL	36.6 (1.1)	37.2 (0.6)	Neg	Neg
	A/WS/33	VR-1520	1.6E + 02 TCID50/mL	34.1 (0.5)	33.3 (0.5)	Neg	Neg
			1.6E + 01 TCID50/mL	37.2 (0.6)	36.8 (0.8)	Neg	Neg
	A/Swine/Iowa/15/1930	VR-333 (ATCC)	8.9E + 03 CEID50/mL	32.1 (0.9)	30.8 (1.2)	Neg	Neg
			8.9E + 02 CEID50/mL	35.2 (1.0)	34.6 (0.4)	Neg	Neg
H3N2 subtype	A/Wisconsin/15/2009	VR-1882 (ATCC)	5.8E + 01 CEID50/mL	33.3 (0.5)	Neg	32.5 (0.7)	Neg
			5.8 E + 00 CEID50/mL	36.2 (0.5)	Neg	37.0 (0.7)	Neg
	A/Victoria/3/75	VR-822 (ATCC)	1.6E + 02 CEID50/mL	32.8 (0.1)	Neg	32.7 (0.2)	Neg
			1.6E + 01 CEID50/mL	36.8 (1.1)	Neg	35.8 (1.0)	Neg
	A/Aichi/2/68	VR-1680 (ATCC)	3.1E + 01 PFU/mL	32.7 (0.3)	Neg	35.0 (0.6)	Neg
			3.1E + 00 PFU/mL	34.9 (0.4)	Neg	35.4 (0.8)	Neg
	A/Alice (recombinant, carries A/England/42/72)	VR-776 (ATCC)	5.0E + 03 TCID50/mL	32.4 (1.9)	Neg	30.3 (2.1)	Neg
			5.0 E + 02 TCID50/mL	34.5 (0.8)	Neg	32.6 (0.6)	Neg
	MRC-2 (recombinant A/England/42/72 and A/PR/8/34 strains)	VR-777 (ATCC)	8.9E + 03 CEID50/mL	31.7 (0.6)	Neg	29.6 (0.5)	Neg
			8.9E + 02 CEID50/mL	37.0 (1.0)	Neg	33.8 (0.7)	Neg
	A/Switzerland/9,715,293/2013	VR-1837 (ATCC)	1.0E + 04 CEID50/mL	32.0 (1.3)	Neg	29.1 (1.1)	Neg
			1.0E + 03 CEID50/mL	36.1 (1.6)	Neg	33.8 (1.6)	Neg
H1N1pdm09 subtype	A/Virginia/ATCC2/2009	VR-1737 (ATCC)	6.1E + 01 PFU/mL	35.5 (2.4)	Neg	Neg	35.0 (1.4)
			6.1E + 00 PFU/mL	Neg	Neg	Neg	Neg
	A/Virginia/ATCC3/2009	VR-1738 (ATCC)	1.8E + 03 PFU/mL	29.0 (1.5)	Neg	Neg	29.3 (1.7)
			1.8E + 02 PFU/mL	35.6 (1.9)	Neg	Neg	33.7 (0.5)
	A/Swine NY/02/2009	0810109CFNHI (Zeptometrix)	1.4E + 01 TCID50/mL	29.9 (0.9)	Neg	Neg	29.2 (0.8)
			1.4E + 00 TCID50/mL	32.2 (0.7)	Neg	Neg	31.5 (0.9)
	A/California/07/2009 NYMC X-179A	VR-1884 (ATCC)	1.4E + 04 CEID50/mL	31.9 (1.3)	Neg	Neg	31.0 (1.4)
			1.4E + 03 CEID50/mL	36.9 (0.9)	Neg	Neg	37.0 (1.5)
	A/Canada/6294/09	0810109CFJHI (Zeptometrix)	1.7E + 01 TCID50/mL	29.4 (0.2)	Neg	Neg	28.7 (0.2)
			1.7E + 00 TCID50/mL	32.7 (1.3)	Neg	Neg	32.2 (1.0)
	A/Netherlands/2629/2009	NR-19823 (ATCC)	1.6E + 02 TCID50/mL	30.4 (0.5)	Neg	Neg	30.2 (0.2)
			1.6E + 01 TCID50/mL	34.5 (0.9)	Neg	Neg	34.5 (1.2)
Non-human Flu A	A/Japan/305/1957 (H2N2)	NR-2775 (ATCC)	3.3E-03 ng/µL	34.7 (0.8)	Neg	Neg	Neg
			3.3E-04 ng/µL	37.8 (n/a)	Neg	Neg	Neg
	A/Korea/426/1968xPuerto Rico/8/1934 (H2N2)	NR-9679 (ATCC)	6.3E-03 ng/µL	33.5 (1.2)	Neg	Neg	Neg
			6.3E-04 ng/µL	36.1 (1.4)	Neg	Neg	Neg
	A/Duck/Singapore/645/1997 (H5N3)	NR-9682 (ATCC)	2.5E-03 ng/µL	33.1 (0.9)	Neg	Neg	Neg
			2.5E-04 ng/µL	34.5 (0.8)	Neg	Neg	Neg
	A/Chicken/Germany/N/49 (H10N7)	NR-2765 (ATCC)	6.8E-03 ng/µL	32.3 (1.4)	Neg	Neg	Neg
			6.8E-04 ng/µL	37.0 (0.4)	Neg	Neg	Neg

SD = Standard Deviation. See Supplementary file 3 for further details on QIAstat-Dx Respiratory SARS-CoV-2 cartridges.

## Results

Bioinformatic screening analysis showed high homology between the generic Influenza A assay (no subtype) included in the QIAstat-Dx Respiratory SARS-CoV-2 Panel and all analyzed 34 Influenza A (Supplementary file 1). Mismatches were low in frequency and located in non-critical positions among the oligonucleotide sequences, rendering a positive amplification prediction by the generic Influenza A assay when testing any of the described (H1)v, H5, H9 swine and G4 EA strains.

On the contrary, detection by the Influenza A subtypes was predicted unequal. In all cases, H3 subtype detection was predicted as negative, and no further laboratory confirmation was required in any strain. This H3 negative result comes from the presence of several mismatches generating no mapping of the primers among genome fragments. In case of H1 subtypes detection (seasonal H1 and H1N1pdm09), QIAstat-Dx oligonucleotides were able to map in all (H1)v, H9N2, and G4-avian like strains, but not for H5N8 genomic sequences. Since Influenza A H5N8 was predicted to be negative for all three subtypes detection by the QIAstat-Dx system, this Influenza A strain is expected to be detected as Influenza A with no subtype discrimination and no further analyses was required.

Based on low presence and non-critical position of mismatches, final Flu A strains selected to be tested in laboratory were (H1)v, H9N2, and G4-avian like genomes against the generic Flu A detection and the two H1 (seasonal and pandemic) assays of the QIAstat-Dx Respiratory SARS-CoV-2 panel (as previously mentioned, the H3 targeted region was considered as negative with several mismatches among all oligonucleotides in the assay). Laboratory testing was focused only in those three RT-qPCR assays (generic Flu A, seasonal H1 and H1N1pdm09 subtypes) using simulated (artificial genomic fragments) Influenza A H1, H9 and G4 EA strains (Table 1 and Supplementary file 1).

All 17 sample combinations covering the complete genetic variability observed among the 33 H1N1, H9N2 and G4 EA strains corroborated bioinformatic prediction with Influenza A positive with no subtype discrimination (Table 1). None of the seasonal subtype assays tested experimentally (H1 and H1N1pdm09) in the QIAstat-Dx Respiratory SARS-CoV-2 Panel presented a cross-reaction with the zoonotic H9 or H1 (including the new G4 genetic cluster). Those results demonstrate the excellent specificity of the QIAstat-Dx Respiratory SARS-CoV-2 Panel with the ability to discriminate only human-specific IAV H1 strains. The presence of EA surface genes (HA and NA genes) of the swine G4 lineage^1^ facilitate the discrimination from human H1 or H1N1pdm09 subtypes.

Based on results, additional testing was done with available commercial Influenza A strains in order to give more clarity on QIAstat-Dx Respiratory SARS-CoV-2 Panel specificity against human and non-human Influenza A strains. Two serial ten-fold dilutions were tested to evaluate Ct correlation between generic Flu A and subtype detection (Table 2 and Supplementary file 3). As a result, all strains corresponding to human subtypes were detected by the generic Flu A design and the corresponding subtype at the same level. Ct values of the two detections were similar, and same Ct shift has been observed for the majority of strains (See Table 2 and Supplementary file 3). As expected, all four non-human Influenza A strains were also detected with no subtype discrimination.

## Discussion

Although the current reports show low incidence of zoonotic Flu A H1 or H9 cases, and H5 cases have reported by first time in humans in latest 2020, it is important to remain vigilant of potentially infective Influenza strains from other species and be prepared to take action in the event of a public health threat^7,8,10^. Particularly in the context of COVID-19 pandemic, the report of a new variant of Influenza A H1 spreading in pigs in China (G4 EA H1N1pdm09-like) described to have high pandemic potential, has generated a reaction from different sources^11,18^. To cover the possibility of a spillover from other species, the overall detection strategy of Influenza A in the QIAstat-Dx Respiratory SARS-CoV-2 Panel is meant to specifically detect and differentiate the human seasonal H1, the human seasonal H3 and the H1N1pdm09 subtypes while providing a generic Influenza A-no subtype assay.

Results demonstrated that bioinformatic analysis showed high homology between the generic Influenza A assay included in the QIAstat-Dx Respiratory SARS-CoV-2 Panel and all screened 34 Influenza A strains. Those results were expected since the generic Influenza A design targets the Matrix gene which is one with highest genetic homology among all IAV strains. It allows discrimination for non-human Flu A strains, with positive detection only for the generic Flu A assay included in the QIAstat-Dx system.

In addition, QIAstat-Dx system provided a clear Ct correlation (Table 2) among dilutions for all human Flu A strains by both generic and subtype assays at low viral concentrations, whereas non-human strains were only detected by the generic Flu A assay with no discrimination. In some cases (Supplementary file 3), discrepancies among replicates were observed with only positive one of the two assays for the human strains. The low viral concentration present in tested samples was selected at Limit of Detection of the QIAstat-Dx Respiratory SARS-CoV-2 assay for a better characterization. In cases of more concentrated samples, an early Ct value for the generic Flu A assay with no correlated positive subtype detection indicates that any non-seasonal H1, non-seasonal H3 or non-H1N1pdm09 Flu A strain is present in the sample. This detection strategy using a generic Influenza A assay fulfils the double function of confirmation of the human-specific subtypes detection while also allowing the potential detection of non-human IAVs.

Results of this study support that the generic Influenza A assay in the QIAstat-Dx Respiratory SARS-CoV-2 Panel is predicted to detect the recorded zoonotic variants such as avian IAVs subtypes A(H5N8) and A(H9N2) and swine IAVs subtypes A(H1N1) and discriminate them from the common strains infecting humans. Based on genetic variability among Flu A subtypes, same results can be extrapolated to other IAVs subtypes as A(H5N1), A(H7N9), A(H1N2) and A(H3N2)^19^.

Here we confirm experimentally that the newly emergent swine Influenza A G4 EA H1N1pdm09-like viruses reported by Sun H. and colleagues^6^, together with those H1N1, H5N8 and H9N2 Influenza A strains detected in humans by 2020, would be detected by the QIAstat-Dx Respiratory SARS-CoV-2 Panel yielding a positive generic Influenza A result and negative result for specific human H3, H1 and H1N1pdm09 subtypes. This contrasts with the report by the CDC of the detection of this potentially pandemic emergent swine G4 strain as seasonal H1N1 positive result using the Influenza Virus Real-time RT-PCR Influenza A (H1/H3/H1N1pdm09) Subtyping panel^11^.

## Conclusions

The results we report supports the overall Influenza A detection strategy of the QIAstat-Dx Respiratory SARS-CoV-2 Panel allowing human-specific Influenza A subtype detection as well as non-human IAVs. Therefore, in the event that the G4 swine EA IAV adaptation causes human-to-human transmission, the QIAstat-Dx Respiratory SARS-CoV-2 Panel could be a useful tool to differentially diagnose zoonotic strains from the seasonal IAVs commonly affecting humans.

Overall, these findings also underline the utility of syndromic testing solutions to provide a differential diagnosis for ILI patients contributing to endemic IAV prevention but also as a key tool for preparedness for potentially pandemic IAV strains of animal origin. With this regard, it will be key to remain vigilant to the new strains to ensure that syndromic diagnostic solutions like the QIAstat-Dx Respiratory SARS-CoV-2 Panel keep their utility for detecting these new strains.

## Supplementary Information

Supplementary Information 1.

Supplementary Information 2.

Supplementary Information 3.

## Data Availability

The authors confirm that the data supporting the findings of this study are available within the article [and/or] its Additional files. All accession numbers from Data bases used in this study can be found in Supplementry file 1. Commercial sequences used for the laboratory testing can be found in Supplementry file 2. Raw data obtained from cartridge workflow for analytical strains analyzed can be found in Supplementry file 3. Primers included in the QIAstat-Dx SARS-CoV-2 Respiratory Panel for Flu A detection are intellectual property of QIAGEN company. They are not necessary for the understanding of this scientific manuscript and to replicate the experiments.

## Abbreviations

Ct: Cycle threshold
CVV: Candidate vaccine virus
EA: Eurasian
H1N1pdm09: H1N1 pandemic 2009
IAV: Influenza A virus
ILI: Influenza-like illness
RT-qPCR: Retrotranscriptase quantitative PCR
SARS-CoV-2: Severe acute respiratory syndrome coronavirus 2
SIV: Swine influenza virus
TR: Triple-reassortant
WHO: World Health Organization
